# The Japan Statin Treatment Against Recurrent Stroke (J-STARS): A Multicenter, Randomized, Open-label, Parallel-group Study

**DOI:** 10.1016/j.ebiom.2015.08.006

**Published:** 2015-08-06

**Authors:** Naohisa Hosomi, Yoji Nagai, Tatsuo Kohriyama, Toshiho Ohtsuki, Shiro Aoki, Tomohisa Nezu, Hirofumi Maruyama, Norio Sunami, Chiaki Yokota, Kazuo Kitagawa, Yasuo Terayama, Makoto Takagi, Setsuro Ibayashi, Masakazu Nakamura, Hideki Origasa, Masanori Fukushima, Etsuro Mori, Kazuo Minematsu, Shinichiro Uchiyama, Yukito Shinohara, Takenori Yamaguchi, Masayasu Matsumoto, for the J-STARS collaborators

**Affiliations:** a Department of Clinical Neuroscience and Therapeutics, Hiroshima University Graduate School of Biomedical and Health Sciences, Hiroshima, Japan; b Foundation for Biomedical Research and Innovation Translational Research Informatics Center, Kobe, Japan; c Hiroshima City Rehabilitation Hospital, Hiroshima, Japan; d Stroke Center, Kinki University, Osakasayama, Japan; e Department of Neurological Surgery, Matsuyama Shimin Hospital, Matsuyama, Japan; f National Cerebral and Cardiovascular Center, Suita, Japan; g Department of Neurology, Tokyo Women's Medical University School of Medicine, Tokyo, Japan; h Department of Neurology, Iwate Medical University, Morioka, Japan; i Department of Neurology, Tokyo Saiseikai Central Hospital, Tokyo, Japan; j Seiai Rehabilitation Hospital, Fukuoka, Japan; k Division of Biostatistics and Clinical Epidemiology, University of Toyama Graduate School of Medicine and Pharmaceutical Sciences, Toyama, Japan; l Department of Behavioral Neurology and Cognitive Neuroscience, Tohoku University Graduate School of Medicine, Sendai, Japan; m Clinical Research Center, International University of Health and Welfare, Center for Brain and Cerebral Vessels, Sanno Hospital and Sanno Medical Center, Tokyo, Japan; n Federation of National Public Service Personnel Mutual Aid Associations Tachikawa Hospital, Tokyo, Japan

**Keywords:** Statin, Ischemic stroke, Hemorrhagic stroke, Atherothrombotic infarction, Cholesterol

## Abstract

**Background:**

Although statin therapy is beneficial for the prevention of initial stroke, the benefit for recurrent stroke and its subtypes remains to be determined in Asian, in whom stroke profiles are different from Caucasian. This study examined whether treatment with low-dose pravastatin prevents stroke recurrence in ischemic stroke patients.

**Methods:**

This is a multicenter, randomized, open-label, blinded-endpoint, parallel-group study of patients who experienced non-cardioembolic ischemic stroke. All patients had a total cholesterol level between 4.65 and 6.21 mmol/L at enrollment, without the use of statins. The pravastatin group patients received 10 mg of pravastatin/day; the control group patients received no statins. The primary endpoint was the occurrence of stroke and transient ischemic attack (TIA), with the onset of each stroke subtype set to be one of the secondary endpoints.

**Finding:**

Although 3000 patients were targeted, 1578 patients (491 female, age 66.2 years) were recruited and randomly assigned to pravastatin group or control group. During the follow-up of 4.9 ± 1.4 years, although total stroke and TIA similarly occurred in both groups (2.56 vs. 2.65%/year), onset of atherothrombotic infarction was less frequent in pravastatin group (0.21 vs. 0.64%/year, p = 0.0047, adjusted hazard ratio 0.33 [95%CI 0.15 to 0.74]). No significant intergroup difference was found for the onset of other stroke subtypes, and for the occurrence of adverse events.

**Interpretation:**

Although whether low-dose pravastatin prevents recurrence of total stroke or TIA still needs to be examined in Asian, this study has generated a hypothesis that it may reduce occurrence of stroke due to larger artery atherosclerosis.

**Funding:**

This study was initially supported by a grant from the Ministry of Health, Labour and Welfare, Japan. After the governmental support expired, it was conducted in collaboration between Hiroshima University and the Foundation for Biomedical Research and Innovation.

## Highlights

• 1578 patients with non-cardioembolic ischemic stroke were randomly assigned to pravastatin group or control group.
• Total stroke and TIA similarly occurred in both groups (2.56 vs. 2.65%/year).
• Atherothrombotic infarction onset was less in pravastatin group (0.21 vs. 0.64%/year, adjusted hazard ratio 0.33).

Although statin therapy is beneficial for the prevention of initial stroke, the benefit for recurrent stroke and its subtypes remains to be determined. This study examined stroke preventive effect of statin on 1578 patients who were assigned to pravastatin group (10 mg/day) or to control group. After 4.9 years of follow-up, although occurrence of total stroke and TIA was not different by the use of pravastatin, occurrence of atherothrombotic infarction was suppressed in the pravastatin group, raising a hypothesis that statins may reduce occurrence of stroke due to larger artery atherosclerosis.

## 1 Introduction

3-Hydroxy-3-methylglutaryl-coenzyme A (HMG-CoA) reductase inhibitors, referred as statins, are widely used to improve serum lipid profiles. In addition to the established value for coronary protection, statins are thought to be beneficial for stroke prevention. Indeed, statin use was associated with 19 to 46% reduction of stroke risk (Pearson, 1998; Plehn et al., 1999; White et al., 2000; Sever et al., 2003; Kushiro et al., 2009; Nomura et al., 2015). However, these findings were derived from patients without prior stroke, and such preventive effect is less robust for patients with occurred stroke. For instance, in the Stroke Prevention by Aggressive Reduction in Cholesterol Levels (SPARCL) trial, the use of atorvastatin was associated with 16% reduction in the risk for recurrent stroke (Amarenco et al., 2006). Also, a meta-analysis of 8 studies demonstrated that statin therapy has only a marginal effect to reduce occurrence of subsequent stroke in patients with prior stroke or transient ischemic stroke (TIA) (Manktelow and Potter, 2009).

Indeed, stroke is a heterogeneous disease with different etiologies, with or without underlying arterial pathologies. Thus, the benefits of statins may be different depending on the subtypes of stroke. For instance, given the structural difference between major cerebral arteries and the perforating branches, the effects of statins can differ between atherothrombotic and lacunar infarctions. Moreover, the use of statins might increase the risk of hemorrhagic stroke (Amarenco et al., 2006; Collins et al., 2004; Boekholdt et al., 2014). Nevertheless, the majority of prior studies defined stroke as a whole, with no distinction between subtypes. Also, although the current international guidelines uniformly recommend the use of statins for secondary stroke prevention (European Stroke Organisation Executive Committee and ESO Writing Committee, 2008; Usherwood, 2013; Kernan et al., 2014), prevalence of lacunar infarction and cerebral hemorrhage is substantially higher in Asian than in Caucasian, requiring further studies to determine whether such guidelines are readily applicable to Asian.

Thus, this study examined whether pravastatin, a traditional statin widely prescribed in the clinic, reduces recurrence of stroke and the respective subtypes in non-cardioembolic stroke patients. Also, whether the use of pravastatin favorably impacts on the occurrence of other vascular events and stroke-related functional outcome was explored.

## 2 Methods

### 2.1 Patients

The design and baseline data of this prospective randomized, open-labeled, blinded-endpoint (PROBE) study was reported previously (Nagai et al., 2014). Briefly, patients aged 45 to 80 years with a history of non-cardioembolic ischemic stroke within the preceding one month to three years were enrolled from 123 centers, between March 2004 and February 2009. All patients had a total cholesterol level between 4.65 and 6.21 mmol/L (180 to 240 mg/dL) at enrollment, without use of statins. The major exclusion criteria included cerebral infarction of determined rare etiology, infarction associated with catheterization or surgery, and preferred use of statins for the treatment of co-morbid coronary artery disease.

This study (NCT00221104) was conducted under the health insurance system of Japan, in accordance with the Declaration of Helsinki and the Ethical Guidelines on Clinical Studies of the Ministry of Health, Labour and Welfare of Japan. Also, this study was approved by the institutional review board of each participating center, and written informed consent was obtained from all patients.

### 2.2 Procedures

Patients were enrolled via a web-based registration and follow-up system provided by the data center, which automatically judged eligibility of each patient and randomly assigned them to pravastatin (10 mg/day) or control group. Of note, given the putative link between statin use and hemorrhagic stroke (Nakamura et al., 2006), and under higher prevalence of cerebral hemorrhage in Japanese (Ikeda et al., 2014), dose of 10 mg/day was chosen, which is the approved standard dose in the nation. In the pravastatin group, the administration was initiated within 1 month after randomization, and the treatment was continued until final observation. Diet and exercise therapies were reinforced when the total cholesterol levels consistently exceeded 6.21 mmol/L (240 mg/dL) at routine clinical visits. Increase of pravastatin dose or addition of other non-statin drugs (such as ion exchange resin, eicosapentaenoic acid, and ezetimibe) was allowed only when such reinforcements were insufficient. Even under such conditions, use of other statins (such as simvastatin and atorvastatin) was prohibited. In the control group, administration of any statin was prohibited, although use of other non-statin drugs was allowed when necessary.

After randomization, patients were followed up at 2 and 6 months, and annually until the study completion. When patient underwent recurrent stroke or other vascular events, such event information was sent to the data center, and managed by dedicated data managers. Brain MRI or CT imaging was performed as previously described (Nagai et al., 2014), so was the measurement of total cholesterol, low density lipoprotein (LDL) cholesterol, triglyceride, and high density lipoprotein (HDL) cholesterol. Treatment compliance was monitored at every clinical visit.

The primary endpoint was the onset of stroke and TIA. Secondary endpoints were the onset of each stroke subtype, myocardial infarction, vascular accident, death, hospitalization, dependence in activities of daily living (modified Rankin Scale, mRS), degree of disability (Barthel Index, BI), onset of dementia, and severity of cognitive impairment. Particularly, stroke and other vascular events were adjudicated by the central event evaluation committees, organized by 4 neurologists for stroke events and 3 cardiologists for cardiac events. The committees were annually held during the study period, in which each event was carefully reviewed based on reports submitted from the participating centers, in a manner strictly blinded from the allocated group information. Also, each event was evaluated in accordance with the predefined definition, the details of which were previously reported (Nagai et al., 2014). For the adjudication of events, the final decision was made by consensus of all committee members. Additionally, correctness of all event adjudication was verified by a local neurologist or cardiologist in each participating center, by collating it with the patient medical records. Dementia was diagnosed by the diagnostic and statistical manual of mental disorders-IIIR criteria. Severity of cognitive impairment was assessed by the clinical dementia rating (CDR) score and the mini-mental state examination (MMSE) score.

### 2.3 Statistical Analysis

In accordance with the intention-to-treat (ITT) principle, the efficacy analysis set was defined as ITT population, including all randomized patients. The safety analysis set included patients who received at least one dose of study drug in the pravastatin group and all patients who were assigned to the control group. Also, the per protocol set was defined by excluding patients with no evaluation of primary endpoint and by additionally excluding patients who took less than 1/4 of pravastatin (as averaged from respective clinical visits) in the pravastatin group and those who took any kind of statins in the control group. The cumulative incidences of time to the first event were estimated by the Kaplan–Meier method. The cumulative incidence curves for the two groups were compared by log-rank test adjusted for the stratification factors at randomization: i.e., stroke subtype (atherothrombotic infarction vs. others), high blood pressure (≥ 150/90 mm Hg vs. not), and diabetes mellitus (absence vs. presence). The Cox proportional hazard model was used to estimate the hazard ratio (HR) and the 95% confidence interval (CI) by adjusting such stratification factors. Changes of mRS, BI, CDR, and MMSE from the baseline were compared by mixed-effects model with repeated measurements (MMRM) with visits defined as fixed effect and baseline values as covariates. In patients without dementia at enrollment, occurrence of dementia was compared between the groups by χ^2^ test. The levels of total cholesterol, LDL cholesterol, triglyceride and HDL cholesterol, and blood pressures were also compared by MMRM.

All analyses were predefined in the statistical analysis plan before the database lock in September 2014, and were conducted using SAS version 9.3 (Cary, NC, USA). Data were expressed as the mean ± standard deviation (SD) for continuous variables and as frequencies and percentages for discrete variables, unless specifically mentioned. The level of significance was set at p < 0.05 (2-tailed).

### 2.4 Role of the Funding Source

This study was initially supported by grants (H14-023, H15-020, H16-003, H17-004) from the Ministry of Health, Labour and Welfare, Japan. After the governmental support expired, it was conducted in collaboration between Hiroshima University Graduate School of Biomedical and Health Sciences and the Foundation for Biomedical Research and Innovation. The latter organization receives unconditional research grants from several pharmaceutical companies, including DAIICHI SANKYO CO., LTD., which commercializes pravastatin. However, the company was not involved in the design and execution of this study. Also, the company did not provide pravastatin for this study and has not reviewed the current manuscript.

## 3 Results

**Fig. 1:**
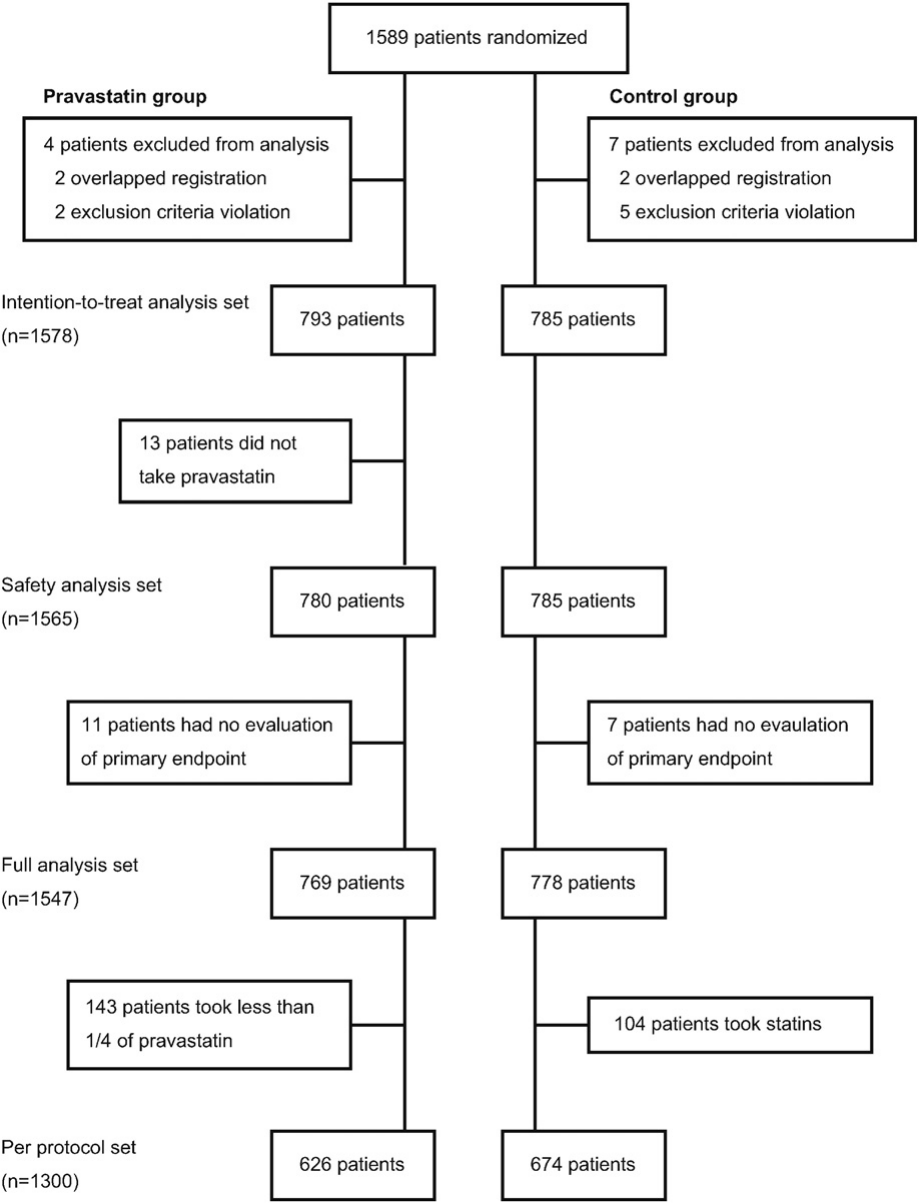
Trial profile.

The 1589 patients were randomly allocated to the pravastatin or control group (Fig. 1). However, due to ineligibility found after randomization or overlapped registration, 4 patients in the pravastatin group and 7 patients in the control group were excluded, resulting in 1578 patients (793 in pravastatin group, 785 in control group) for intention-to-treat analysis. Also, in the pravastatin group, 13 patients (1.6%) did not take pravastatin and 11 patients (1.4%) had no evaluation of primary endpoint. In the control group, 7 patients (0.9%) had no evaluation of primary endpoint. Also, 143 patients (18.0%) in pravastatin group took less than 1/4 of prescribed drug, and 104 patients (13.2%) in control group received certain kinds of statins during the follow-up (Supplementary Table S1). Final follow-up rates were 79.3% in the pravastatin group and 80.4% in the control group.

**Fig. 2:**
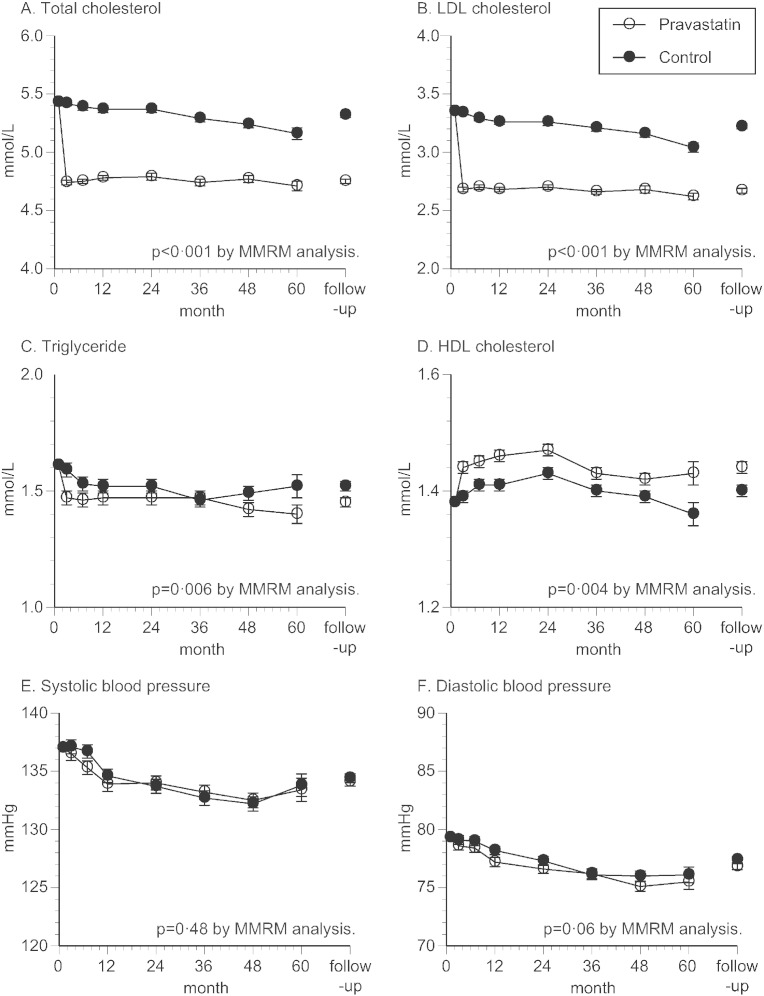
Changes in lipid profile and blood pressure. Changes in the lipid profile and blood pressure were analyzed by mixed-effects model with repeated measurements (MMRM). Open and close circles represent adjusted mean, with standard error expressed by error bars. Levels of total cholesterol (A), low density lipoprotein (LDL) cholesterol (B), and triglyceride (C) were lower in the pravastatin group. Level of high density lipoprotein (HDL) cholesterol was higher in the pravastatin group (D). Systolic blood pressure (E) and diastolic blood pressure (F) were appropriately controlled in the normal ranges in both groups. Mean values (mean ± SE) of total cholesterol during follow-up periods in pravastatin group and control group were 4.75 ± 0.02 vs. 5.32 ± 0.02 mmol/L, LDL cholesterol 2.67 ± 0.02 vs. 3.22 ± 0.02 mmol/L, triglyceride 1.45 ± 0.02 vs. 1.52 ± 0.02 mmol/L, HDL cholesterol 1.44 ± 0.01 vs. 1.40 ± 0.01 mmol/L, systolic blood pressure 134.1 ± 0.38 vs. 134.4 ± 0.38 mm Hg, and diastolic blood pressure 76.8 ± 0.25 vs. 77.4 ± 0.25 mm Hg, respectively.

The baseline characteristics of patients are shown in Table 1, demonstrating no significant difference in parameters between the two groups. Particularly, lipid and blood pressure levels, proportions of stroke subtypes, and use of antiplatelet agents were well balanced between the two groups. During the follow up, total cholesterol, LDL cholesterol, and triglyceride levels were lower in pravastatin group (Fig. 2A–C), whereas HDL cholesterol level was slightly higher in pravastatin group (Fig. 2D). Also, change of systolic and diastolic blood pressure was similar between the two groups (Fig. 2E,F), so was the duration of follow-up (4.86 ± 1.45 vs. 4.93 ± 1.44 years, respectively).

**Fig. 3:**
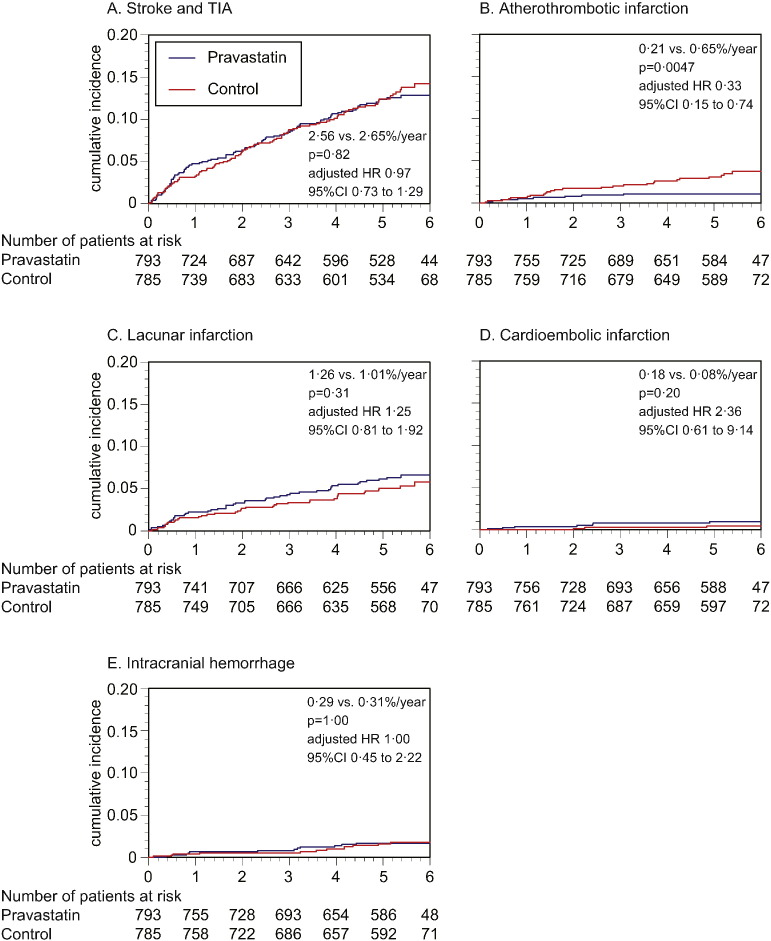
Kaplan–Meier curves for the primary and secondary endpoints. Although stroke and TIA similarly occurred in the pravastatin and control groups (A), occurrence of atherothrombotic infarction was less frequent in the pravastatin group (B). Occurrence of lacunar infarction (C), cardioembolic infarction (D), and intracranial hemorrhage (E) was similar between the two groups. Hazard ratios are adjusted for the stratification factors at randomization: i.e., stroke subtype (atherothrombotic infarction vs. others), high blood pressure (≥ 150/90 mm Hg vs. not), and diabetes mellitus (absence vs. presence).

As the primary endpoint, total stroke and TIA similarly occurred in pravastatin and control group (2.56 vs. 2.65%/year, p = 0.82, adjusted HR 0.97 [95%CI 0.73 to 1.29], Fig. 3A), and the finding was virtually unaffected when TIA was excluded from the analysis (2.35 vs. 2.47%/year, p = 0.74, adjusted hazard ratio 0.95 [95%CI 0.71 to 1.28]). However, occurrence of atherothrombotic infarction was less frequent in the pravastatin group (0.21 vs. 0.65%/year, p = 0.0047, adjusted HR 0.33 [95%CI 0.15 to 0.74], Fig. 3B). Additionally, such trend was present in patients both with atherothrombotic and lacunar infarction at baseline (Appendix Table 2), and appeared to exist in certain subgroups of patients (Appendix Fig. 2). Occurrence of lacunar infarction (Fig. 3C), cardioembolic infarction (Fig. 3D), and intracranial hemorrhage (Fig. 3E) was similar between the two groups, so was the occurrence of myocardial infarction, vascular accidents, death, and hospitalization (Table 2).

**Fig. 4:**
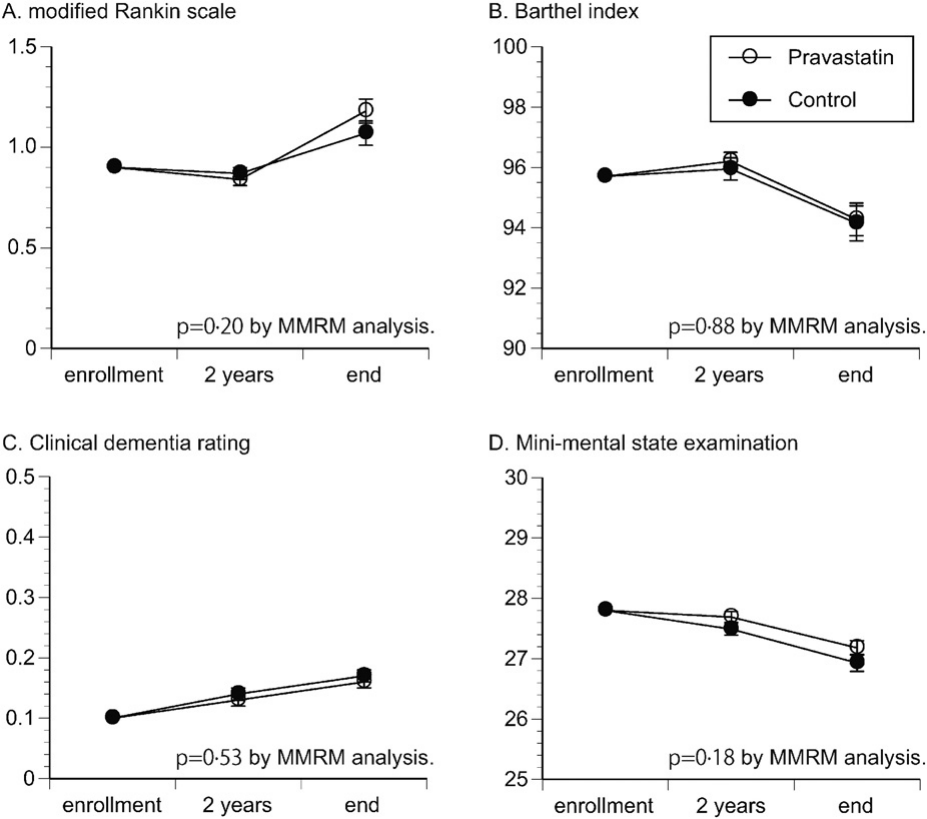
Changes in stroke-related outcomes. Changes in modified Rankin scale score (A), Barthel index score (B), clinical dementia rating score (C), and mini-mental state examination score (D) were analyzed by mixed-effects model with repeated measurements (MMRM). Open and close circles represent adjusted mean, with standard error expressed by error bar.

As stroke-related functional measures, changes of mRS scores and BI score were similar between the two groups (Fig. 4A,B), so was the change of CDR score (Fig. 4C). Decline of MMSE score tended to be mild in the pravastatin group, although the difference was not significant (Fig. 4D). Also, in patients without dementia at enrollment, incidence of newly diagnosed dementia was 4.1% (0.85%/year) in pravastatin group and 4.2% (0.84%/year) in control group (p = 0.94).

Significant intergroup differences were not found in the occurrence of adverse events, including cancer, rhabdomyolysis, and laboratory examinations (Supplementary Table S3).

## 4 Discussion

As previously reported (Nagai et al., 2014), although the sample size was initially set to be 3000, the target number was not achieved and 1589 patients were recruited for this study (Fig. 1), largely due to the narrower window of patient recruitment. Also, several patients were excluded after randomization, resulting in 1578 patients for the intention-to-treat analysis. Randomization was successfully conducted and both group of patients demonstrated similarly well controlled cardiovascular risk factor profiles at enrollment (Table 1), representing a population at lower risk for stroke recurrence. Also, use of anti-platelet agents exceeded 90% in both groups, which could have even reduced the risk for stroke recurrence. Of note, proportions of stroke subtypes were similar between the two groups, with roughly two thirds of patients having lacunar infarction, which was two times higher than reported in SPARCL trial (Amarenco et al., 2009), but similar to the recent report from Japan (Shinohara et al., 2010).

Although the dose of pravastatin (10 mg/day) was lower than used in studies from the Western countries (Shepherd et al., 2002; ALLHAT Officers and Coordinators for the ALLHAT Collaborative Research Group, 2002), it was the approved standard dose in Japan. Indeed, the levels of total cholesterol and LDL cholesterol were substantially reduced and kept in the normal ranges in the pravastatin group (Fig. 2A,B). Also, level of HDL cholesterol was slightly higher in the pravastatin group (Fig. 2D), which could have exerted favorable impact on arteries in this group of patients (Kuwashiro et al., 2012; Nishimura et al., 2013). As a well-defined stroke risk factor, blood pressure level was similarly well controlled in both groups (Fig. 2E,F), making it unlikely that the level exerted significant influence on the recurrence of stroke in either group.

Given the nature of study sample, incidence of recurrent stroke (approximately 2.6%/year in both groups) was roughly half of our initial assumption, but turned out to be similar to the recent report from Japan (Shinohara et al., 2010). As the primary endpoint, although total stroke recurrence was similar between the two groups, patient accrual was insufficient and statistical power was not enough, requiring further studies to adequately address to the intergroup difference. However, even under such conditions, onset of atherothrombotic infarction was clearly less frequent in pravastatin group, whereas no significant difference was found for other stroke subtypes (Fig. 3). This finding may be reasonable, if pravastatin exerted atheroprotective effects on the carotid and major cerebral arteries (Crisby et al., 2001; Byington et al., 1995; Crouse et al., 2007), thus suppressing the progression of underlying atherosclerotic processes. However, how levels of LDL cholesterol or other pleiotropic effects have contributed to the risk reduction cannot be determined from this study. Of note, reduction of LDL cholesterol was roughly 20% in this study, compared to the baseline (Fig. 2B), which may not be an effective reduction for stroke prevention (Boekholdt et al., 2014; Corvol et al., 2003; Amarenco et al., 2004). On the other hand, many studies have suggested pleiotropic effects of statins, including atheromatous plaque stabilization and anti-inflammation (Crisby et al., 2001; Byington et al., 1995; Crouse et al., 2007; Tsuda et al., 1996; Ridker et al., 1999; Albert et al., 2001; Uchiyama et al., 2009), which could have played a role for the suppression of atherothrombotic infarction as found in this study. Concurrent with this study, sub-studies focusing on chronic inflammation (NCT00361699) and carotid atherosclerosis (NCT00361530) were conducted, the results of which would allow for additional elucidation on the relationship between stroke recurrence and statin use.

Additionally, incidence of lacunar infarction was similar between the two groups, or tended to be even higher (not significant) in the pravastatin group (Fig. 3C). On the basis of this finding, pravastatin is not likely to suppress the stroke of small arterial pathologies, as generally referred to “small vessel disease.” However, stroke patients often have underlying cardiovascular complications and risk factors, for which utility of statin treatments should not be undervalued. Of note, although statin treatment could increase the risk of intracranial hemorrhage (Amarenco et al., 2006; Boekholdt et al., 2014), the occurrence was virtually the same between two groups (Fig. 3E). In a meta-analysis by Boekholdt et al., the risk for intracranial hemorrhage was higher in patients with very low LDL cholesterol level (< 1.94 mmol/L) than in patients with moderately low LDL cholesterol level (2.58 to 3.23 mmol/L) (Boekholdt et al., 2014). In the current study, LDL cholesterol level in the pravastatin group was only “moderately low” (2.67 mmol/L, Fig. 2B), and was substantially higher than in SPARCL trial (1.89 mmol/L) in which risk of hemorrhagic stroke was increased (Amarenco et al., 2006).

As stroke-related functional measures, mRS and BI similarly deteriorated in both groups (Fig. 4A,B), with no apparent difference. Also, given the studies suggesting favorable effects of statin on cognitive function, whether change of CDR and MMSE score differs between the two groups was of interest. In the current study, CDR score similarly deteriorated during the time course in both groups (Fig. 4C). However, decline of MMSE score tended to be less in the pravastatin group although the change was not significant (Fig. 4D), which may be in line with a previous meta-analysis, suggesting an effect of statins for mitigating decline of MMSE score (McGuinness et al., 2014). Additionally, incidence of newly diagnosed dementia was relatively low as in prior studies (Forette et al., 1998; Tzourio et al., 2003), with no significant difference between the groups. Further studies are required to define the impact of statin on cognitive function and dementia.

The current study has certain limitations. First, in part because of the restriction of national health insurance system, this study was conducted by the PROBE method, potentially allowing for arbitrariness in the endpoint evaluation. Under such condition, we made every effort to increase accuracy of event adjudication. Particularly, all stroke and other vascular events were reviewed by the dedicated central event evaluation committees in a strictly blinded manner. Also, all events were adjudicated precisely in accordance with the predefined definition (Nagai et al., 2014), to prevent inconsistent judgment between committee members and between occasions. Second, although all stroke and other vascular events were cautiously reviewed and adverse events were eagerly collected, how lack of blinding impacted on reporting from the local centers is not known. Third, because this study was conducted as part of the clinical practice, we could not strictly prohibit the use of statins in the control group. Indeed, in part because of the study demonstrating favorable effects of statins for stroke prevention (Amarenco et al., 2006), more than 10% of patients in the control group took some kinds of statins. Inversely, nearly 20% of patients in the pravastatin group did not take pravastatin or took less than 1/4 of prescribed pravastatin. However, such protocol violations would dilute intrinsic differences between the groups and decrease the likelihood of achieving statistical significance. Under such conditions, the robustness of our analyses was tested for the per protocol analysis set (Fig. 1). By such sensitivity analysis, intergroup difference in the occurrence of atherothrombotic infarction persisted (0.15 vs. 0.55%/year, p = 0.0047, adjusted HR 0.26 [95%CI 0.10 to 0.71]), supporting the reliability of our finding.

## 5 Conclusion

Although whether low-dose pravastatin prevents recurrence of total stroke or TIA still needs to be examined in Asian, this study has generated a hypothesis that it may reduce occurrence of stroke due to larger artery atherosclerosis.

## Author's Contributions

MM is the principal investigator. YN, TK, CY, KK, SI, MF, KM, SU, and MM are responsible for the conception and design of the study. NH, YN, TK, TO, SA, TN, HM, NS, CY, KK, YT, MT, SI, KM, SU, YS, and MM participated in data collection. NH, YN, SA, CY, SI, HO, MF, KM, SU, and MM made the statistical analysis plan. YN, HO, and MF performed the statistical analysis. NH, YN, MN, EM, TY, and MM made data interpretation. NH, YN, and MM designed the figures. NH, YN, TK, CY, and MM made literature search. NH, YN, KK, KM, SU, and MM contributed to draft the report. All authors participated in the finalization of the report.

The following are the supplementary data related to this article

**Supplementary Figs. S1–4:**
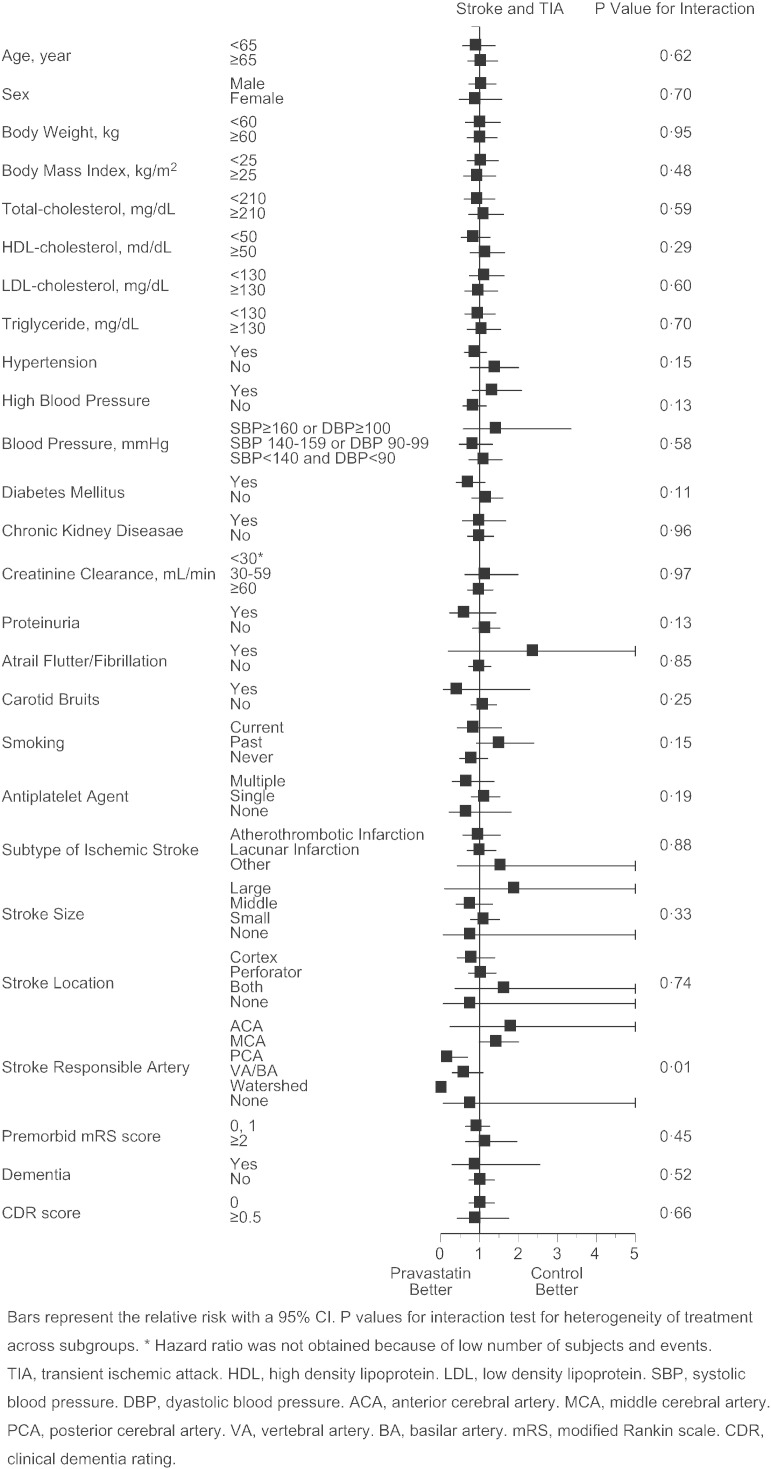

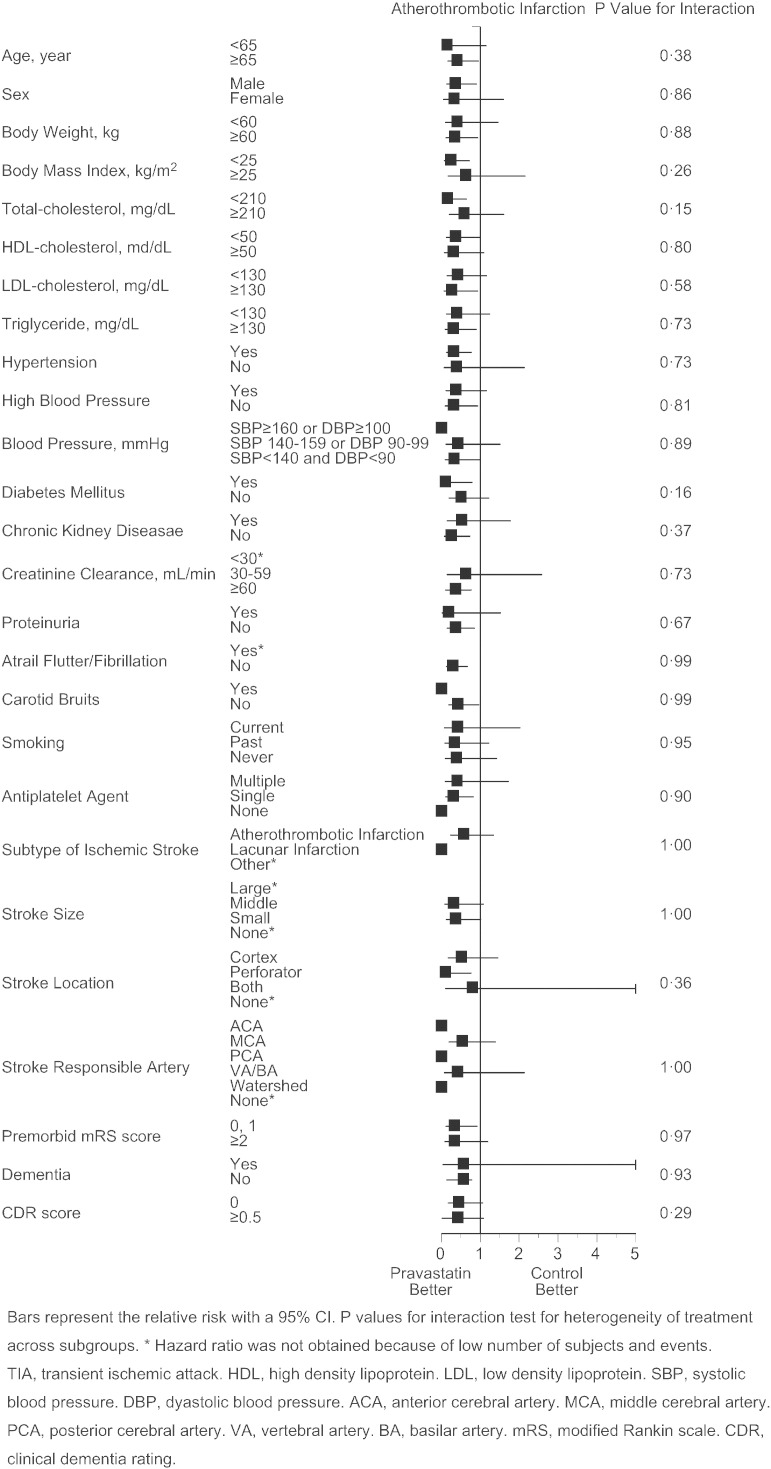

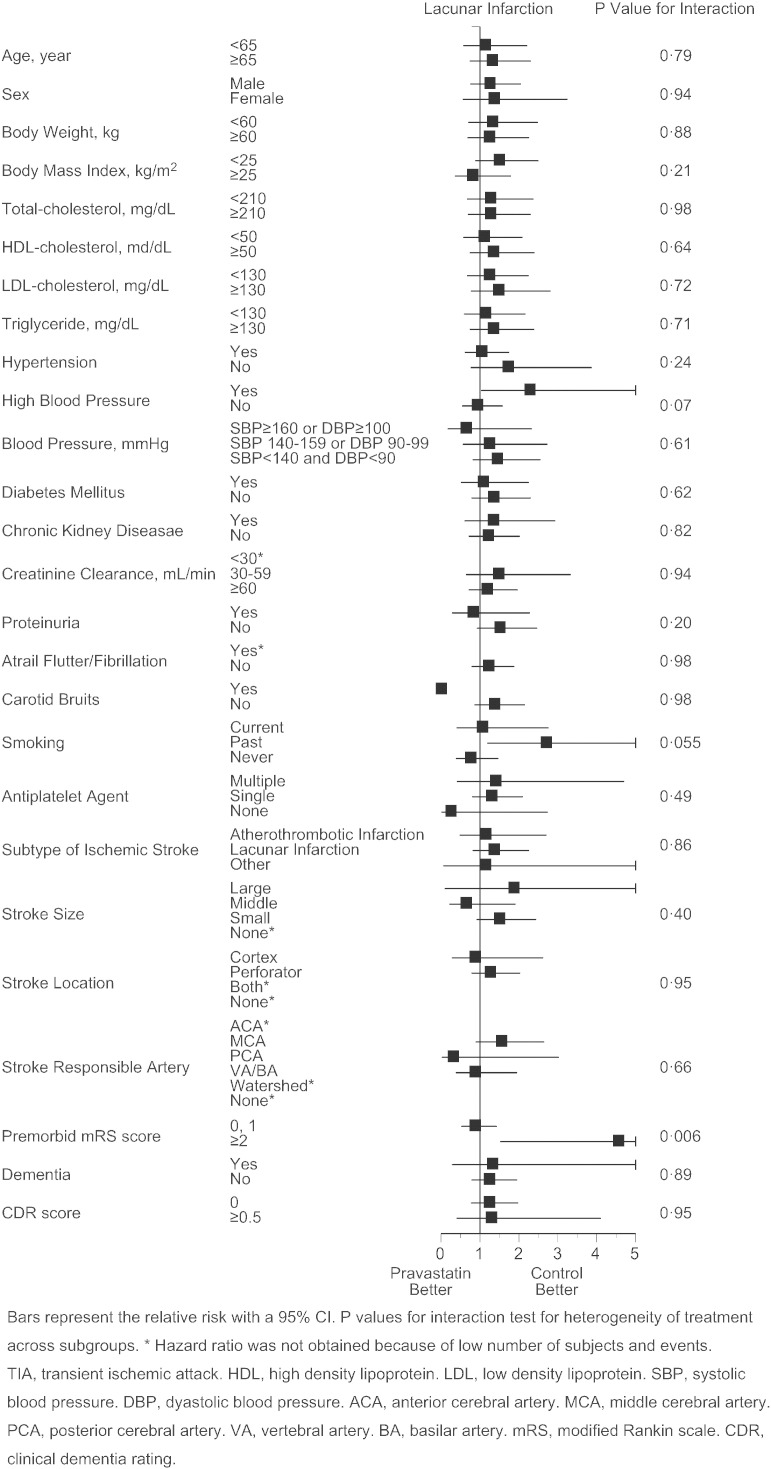

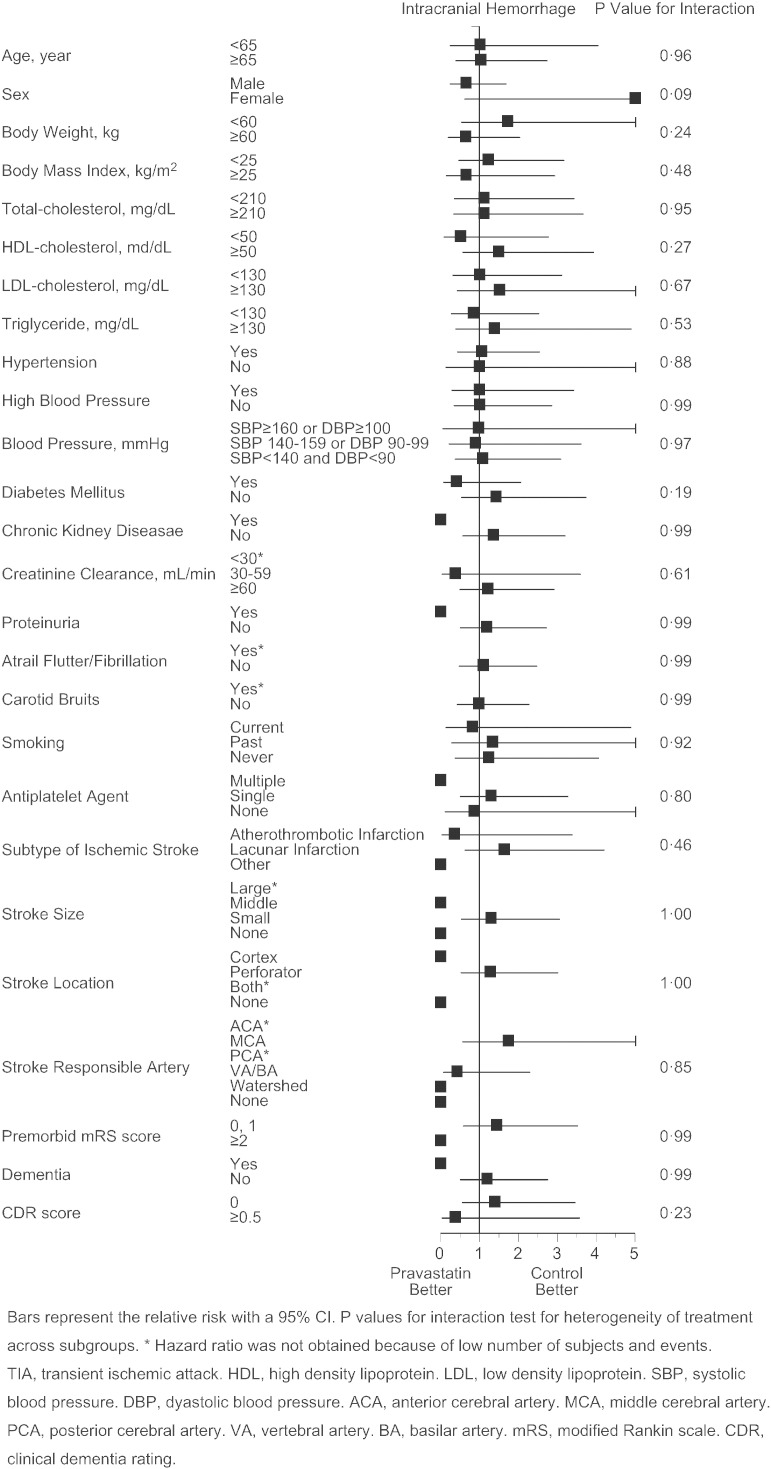
Cox proportional hazards for stroke and TIA (S1), atherothrombotic infarction (S2), lacunar infarction (S3), and intracranial hemorrhage (S4) in pre-defined subgroups. Bars represent the relative risk with a 95%CI. p values for interaction test for heterogeneity of treatment across subgroups. *Hazard ratio was not obtained because of low number of subjects and events. TIA, transient ischemic attack. HDL, high density lipoprotein. LDL, low density lipoprotein. SBP, systolic blood pressure. DBP, diastolic blood pressure. ACA, anterior cerebral artery. MCA, middle cerebral artery. PCA, posterior cerebral artery. VA, vertebral artery. BA, basilar artery. mRS, modified Rankin scale. CDR, clinical dementia rating.

Supplementary tables. Supplementary Table S1. Visit and Medication Compliances. Supplementary Table S2. Occurrence of each stroke subtype by the baseline stroke diagnoses. Supplementary Table S3. Occurrence of Major Adverse Events.

Supplementary material. Appendix. J-STARS Group: Organizational Structure and Participants.

## Figures and Tables

**Table 1:** Baseline characteristics.

	Pravastatin group n = 793	Control group n = 785
Age, years	66.1 ± 8.4	66.4 ± 8.6
Male, n (%)	545 (68.7)	542 (69.0)
Height, cm	160.4 ± 8.8	160.1 ± 8.6
Weight, kg	61.5 ± 10.2	60.7 ± 10.1
Body mass index, kg/m^2^	23.8 ± 3.1	23.6 ± 3.0
Total cholesterol, mmol/L	5.45 ± 0.62	5.42 ± 0.64
HDL cholesterol, mmol/L	1.39 ± 0.41	1.37 ± 0.41
LDL cholesterol, mmol/L	3.35 ± 0.63	3.35 ± 0.64
Triglyceride, mmol/L	1.61 ± 0.85	1.60 ± 0.82
Hypertension, n (%)	596 (75.2)	604 (76.9)
Systolic blood pressure, mm Hg	137.3 ± 17.6	136.9 ± 18.0
Diastolic blood pressure, mm Hg	79.3 ± 11.6	79.4 ± 10.9
Diabetes mellitus, n (%)	185 (23.3)	184 (23.4)
Fasting blood glucose, mmol/L	6.56 ± 2.38	6.49 ± 2.17
Coronary artery disease, n (%)	37 (4.7)	44 (5.6)
Chronic kidney disease, n (%)	195 (24.6)	183 (23.3)
Creatinine, md/dL	0.81 ± 0.21	0.80 ± 0.21
Smoking habit		
Smoker, n (%)	426 (53.7)	420 (53.5)
Non-smoker, n (%)	358 (45.1)	352 (44.8)
Unknown, n (%)	9 (1.1)	13 (1.7)
Use of antiplatelet agents, n (%)	723 (91.2)	715 (91.1)
Ischemic stroke subtype		
Atherothrombotic infarction, n (%)	195 (24.6)	206 (26.2)
Lacunar infarction, n (%)	502 (63.3)	504 (64.2)
Infarction of undetermined etiology, n (%)	96 (12.1)	75 (9.6)

No parameters were significantly different between the pravastatin and control groups.

**Table 2:** Incidence of vascular events other than stroke.

	Event rate, %/year		Adjusted hazard ratio	95% confidence interval	p value
Pravastatin	Control
Myocardial Infarction	0.10	0.18	0.55	0.16 to 1.89	0.34
Vascular Accidents	3.23	3.81	0.85	0.66 to 1.09	0.19
Death	1.11	0.90	1.23	0.79 to 1.93	0.36
Hospitalization	9.25	9.73	1.02	0.85 to 1.22	0.85

Hazard ratios are adjusted for the stratification factors at randomization: i.e., stroke subtype (atherothrombotic infarction vs. others), high blood pressure (≥ 150/90 mm Hg vs. not), and diabetes mellitus (absence vs. presence).
