# Supplementary material for: The Japan Statin Treatment Against Recurrent Stroke (J-STARS): A Multicenter, Randomized, Open-label, Parallel-group Study
Source: eBioMedicine. 2015 Aug 6;2(9):1071–8. doi: 10.1016/j.ebiom.2015.08.006 (PMC4588424; doi:10.1016/j.ebiom.2015.08.006)
Supplement: Supplementary tables. — Supplementary Table S1. Visit and Medication Compliances. Supplementary Table S2. Occurrence of each stroke subtype by the baseline stroke diagnoses. Supplementary Table S3. Occurrence of Major Adverse Events. [file mmc1.pdf]

Supplementary Table S1. Visit and Medication Compliances.

	Month 2	Month 6	Year 1	Year 2	Year 3	Year 4	Year 5	End of follow-up
Pravastatin								
Patients, n	793	781	772	760	731	702	612	793
Actual visits, n	793	780	770	758	728	700	288	685
20 mg pravastatin, n (%)	0	3 (0.4)	1 (0.1)	2 (0.3)	2 (0.3)	4 (0.6)	0	3 (0.4)
15 mg pravastatin, n (%)	0	0	0	0	0	0	0	1 (0.1)
10 mg pravastatin, n (%)	780 (98.4)	731 (93.7)	702 (91.2)	649 (85.6)	596 (81.9)	552 (78.9)	219 (76.0)	492 (71.8)
5 mg pravastatin, n (%)	0	1 (0.1)	2 (0.3)	1 (0.1)	1 (0.1)	2 (0.3)	0	2 (0.3)
No pravastatin, n (%)	13 (1.6)	45 (5.8)	65 (8.4)	106 (14.0)	129 (17.7)	142 (20.3)	69 (24.0)	187 (27.3)
Other statin, n (%)	0	2 (0.3)	3 (0.4)	10 (1.3)	9 (1.2)	11 (1.6)	8 (2.8)	20 (2.9)
Other lipid-lowering drug, n (%)	0	4 (0.5)	6 (0.8)	8 (1.1)	9 (1.2)	9 (1.3)	9 (3.1)	18 (2.6)
Control								
Patients, n	785	780	777	765	731	707	620	785
Actual visits, n	784	777	772	762	730	706	285	694
No lipid-lowering drug, n (%)	667 (85.1)	644 (82.9)	616 (79.8)	557 (73.1)	505 (69.2)	466 (66.0)	179 (62.8)	423 (61.0)
Pravastatin, n (%)	1 (0.1)	4 (0.5)	10 (1.3)	13 (1.7)	18 (2.5)	20 (2.8)	10 (3.5)	25 (3.6)
Other statin, n (%)	11 (1.4)	19 (2.4)	26 (3.4)	34 (4.5)	40 (5.5)	52 (7.4)	26 (9.1)	53 (7.6)
Other lipid-lowering drug, n (%)	95 (12.1)	91 (11.7)	96 (12.4)	98 (12.9)	103 (14.1)	89 (12.6)	31 (10.9)	77 (11.1)

Supplementary Table S2. Occurrence of each stroke subtype by the baseline stroke diagnoses.

	Recurred Stroke			
Baseline Stroke	Atherothrombotic Infarction	Lacunar Infarction	Cardioembolic Infarction	Intracranial Hemorrhage
Atherothrombotic Infarction	8/15	11/10	3/0	1/3
Lacunar Infarction	0/10	35/27	3/3	11/7
Undetermined Etiology Stroke	0/0	1/1	1/0	0/2

Figures represent number of patients (pravastatin group/control group) who developed respective subtypes of recurred stroke.

Supplementary Table S3. Occurrence of Major Adverse Events.

Event Name	Pravastatin n=780	Control n=785
Any adverse event, n (%)	188 (24.1)	164 (20.9)
Any cancer, n (%)	43 (5.5)	40 (5.1)
Other adverse events		
Pneumonia, n (%)	14 (1.8)	11 (1.4)
Fracture, n (%)	12 (1.5)	10 (1.3)
Cataract, n (%)	9 (1.2)	10 (1.3)
Colon polyp, n (%)	9 (1.2)	7 (0.9)
Rhabdomyolysis, n (%)	2 (0.3)	1 (0.1)
Laboratory examination		
AST, > 3.0 x ULN	8 (1.0)	4 (0.5)
ALT, > 3.0 x ULN	6 (0.8)	2 (0.3)
Creatine kinase, > 2.5 x ULN	7 (0.9)	4 (0.5)

Adverse events occurred at the incidence of ≥1.0%, and those specifically related with the studied drug are listed.

ULN, upper limit of normal.

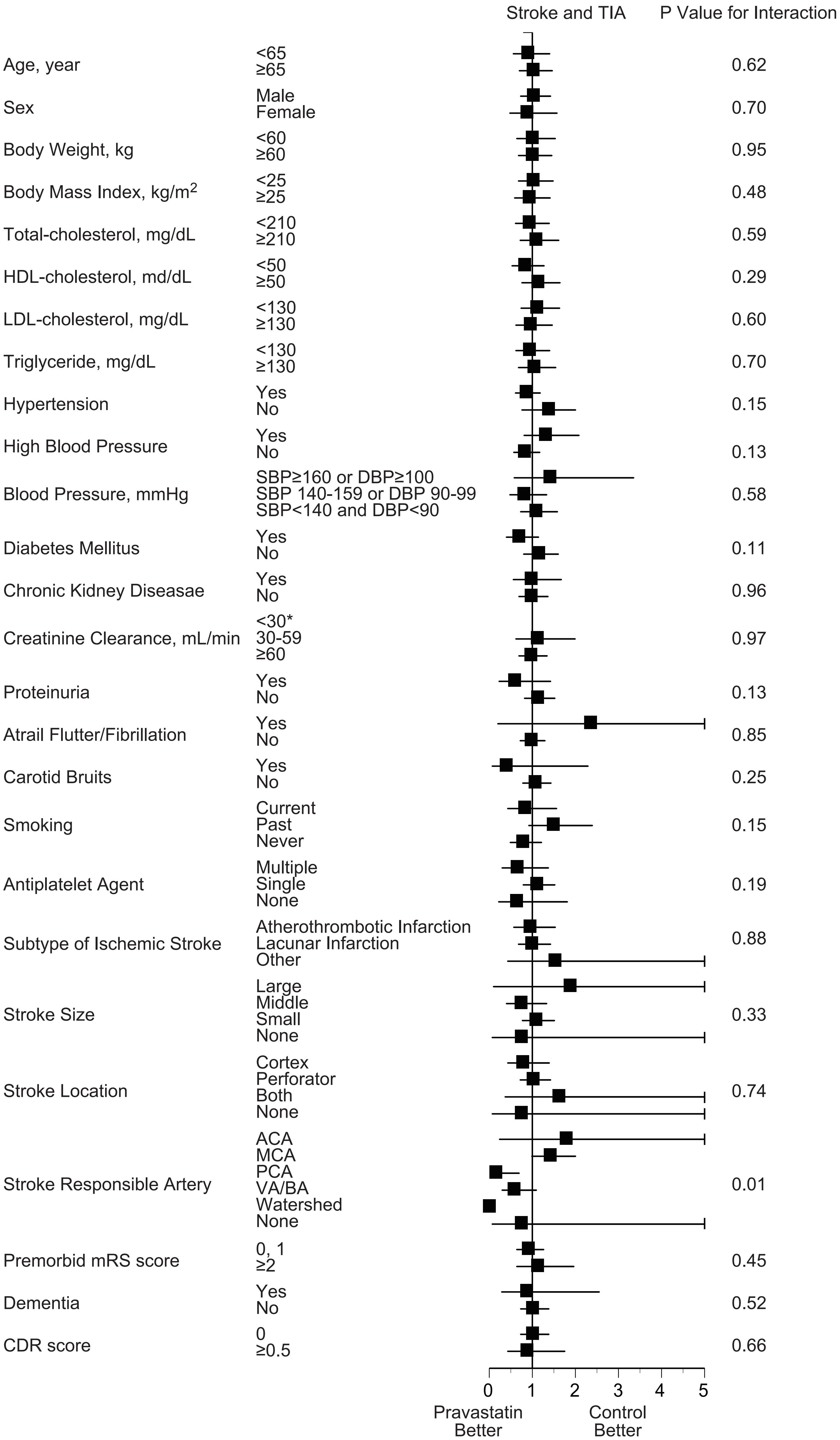

Supplementary Fig. S1. Cox proportional hazards for stroke and TIA in pre-defined subgroups.
Bars represent the relative risk with a 95%CI. P values for interaction test for heterogeneity of treatment across subgroups. *Hazard ratio was not obtained because of low number of subjects and events.
TIA, transient ischemic attack. HDL, high density lipoprotein. LDL, low density lipoprotein. SBP, systolic blood pressure. DBP, diastolic blood pressure. ACA, anterior cerebral artery. MCA, middle cerebral artery. PCA, posterior cerebral artery. VA, vertebral artery. BA, basilar artery. mRS, modified Rankin scale. CDR, clinical dementia rating.

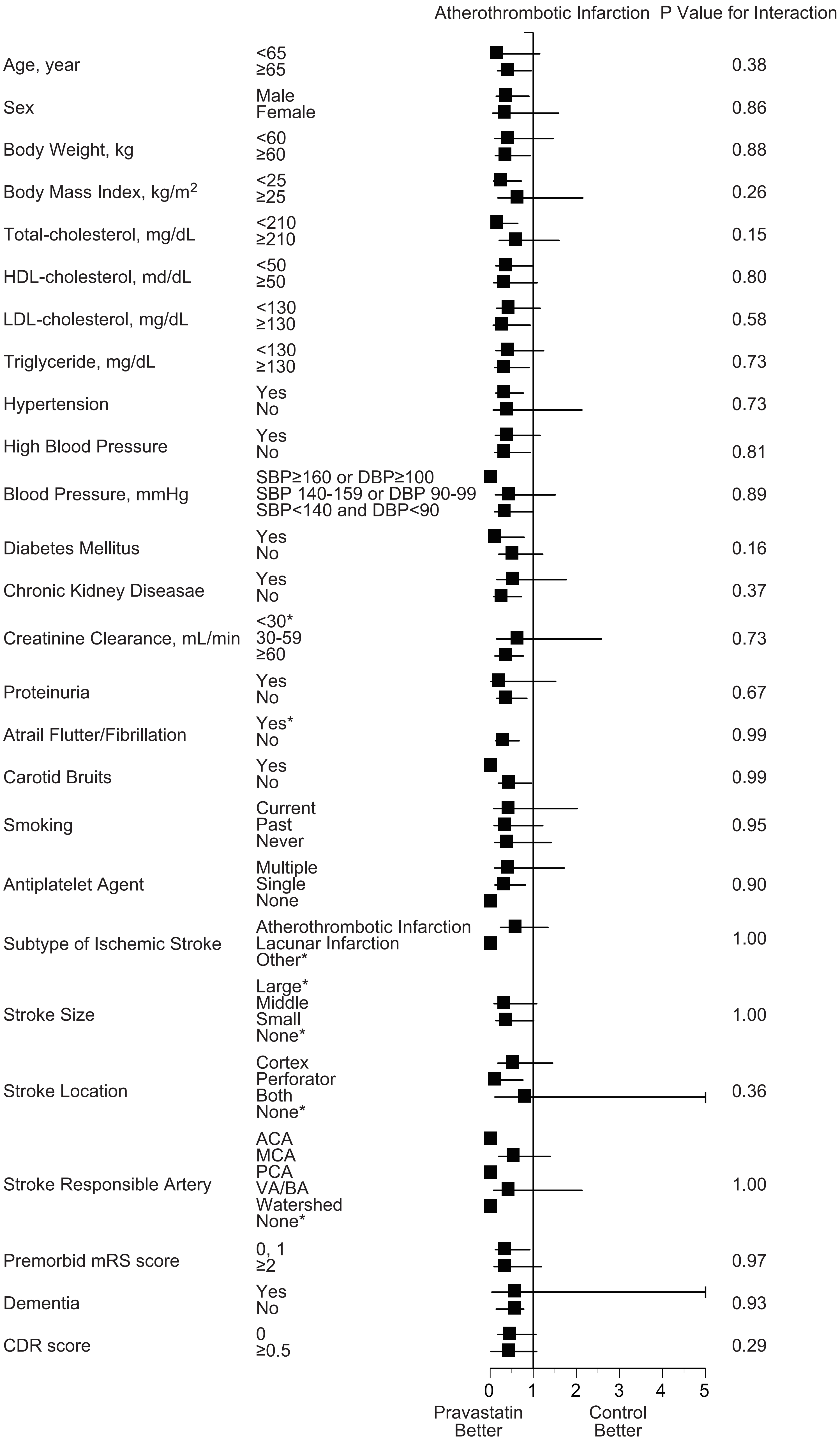

Supplementary Fig. S2. Cox proportional hazards for atherothrombotic infarction in pre-defined subgroups. Bars represent the relative risk with a 95%CI. P values for interaction test for heterogeneity of treatment across subgroups. *Hazard ratio was not obtained because of low number of subjects and events.

TIA, transient ischemic attack. HDL, high density lipoprotein. LDL, low density lipoprotein. SBP, systolic blood pressure. DBP, diastolic blood pressure. ACA, anterior cerebral artery. MCA, middle cerebral artery. PCA, posterior cerebral artery. VA, vertebral artery. BA, basilar artery. mRS, modified Rankin scale. CDR, clinical dementia rating.

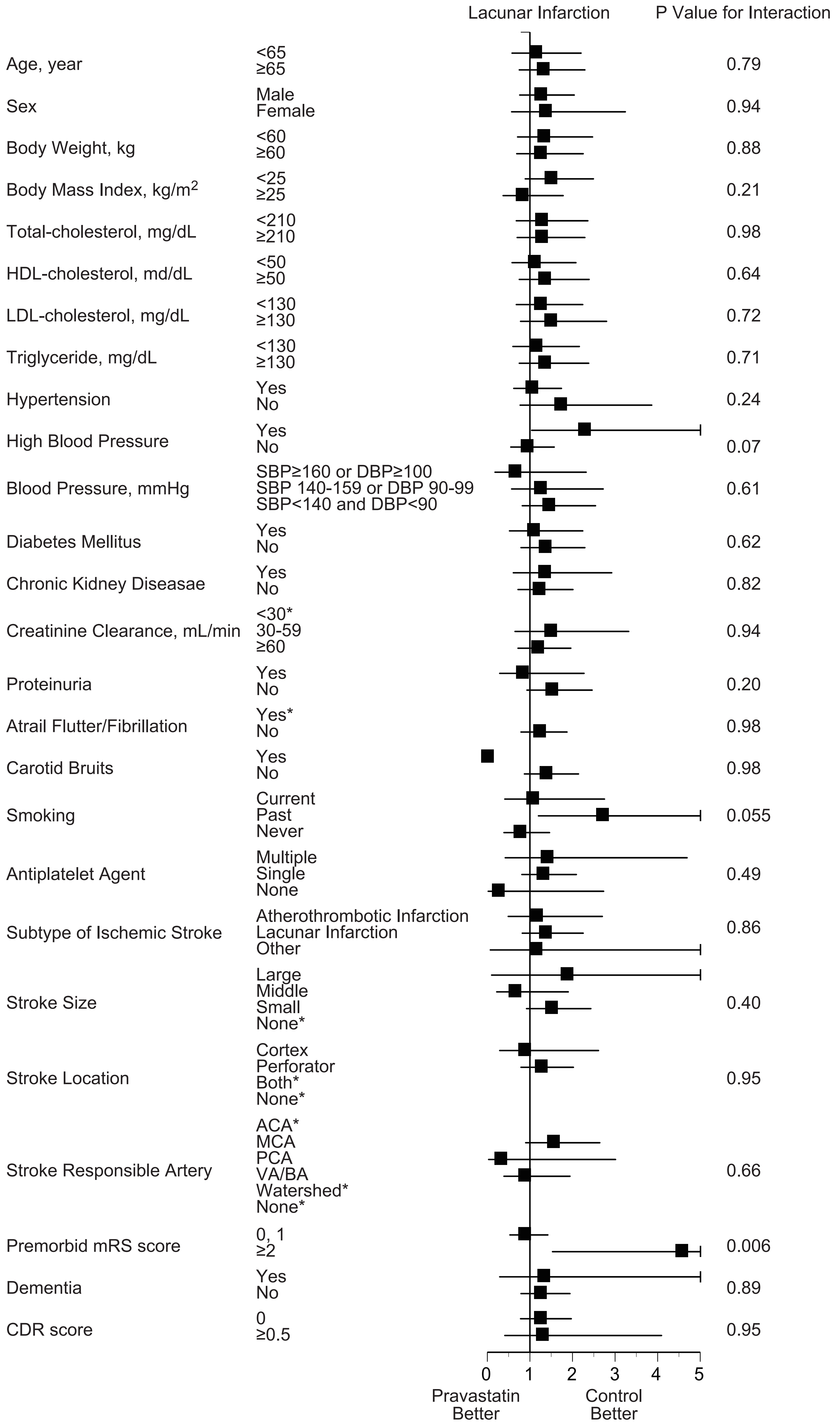

Supplementary Fig. S3. Cox proportional hazards for lacunar infarction in pre-defined subgroups. Bars represent the relative risk with a 95%CI. P values for interaction test for heterogeneity of treatment across subgroups. *Hazard ratio was not obtained because of low number of subjects and events. TIA, transient ischemic attack. HDL, high density lipoprotein. LDL, low density lipoprotein. SBP, systolic blood pressure. DBP, diastolic blood pressure. ACA, anterior cerebral artery. MCA, middle cerebral artery. PCA, posterior cerebral artery. VA, vertebral artery. BA, basilar artery. mRS, modified Rankin scale. CDR, clinical dementia rating.

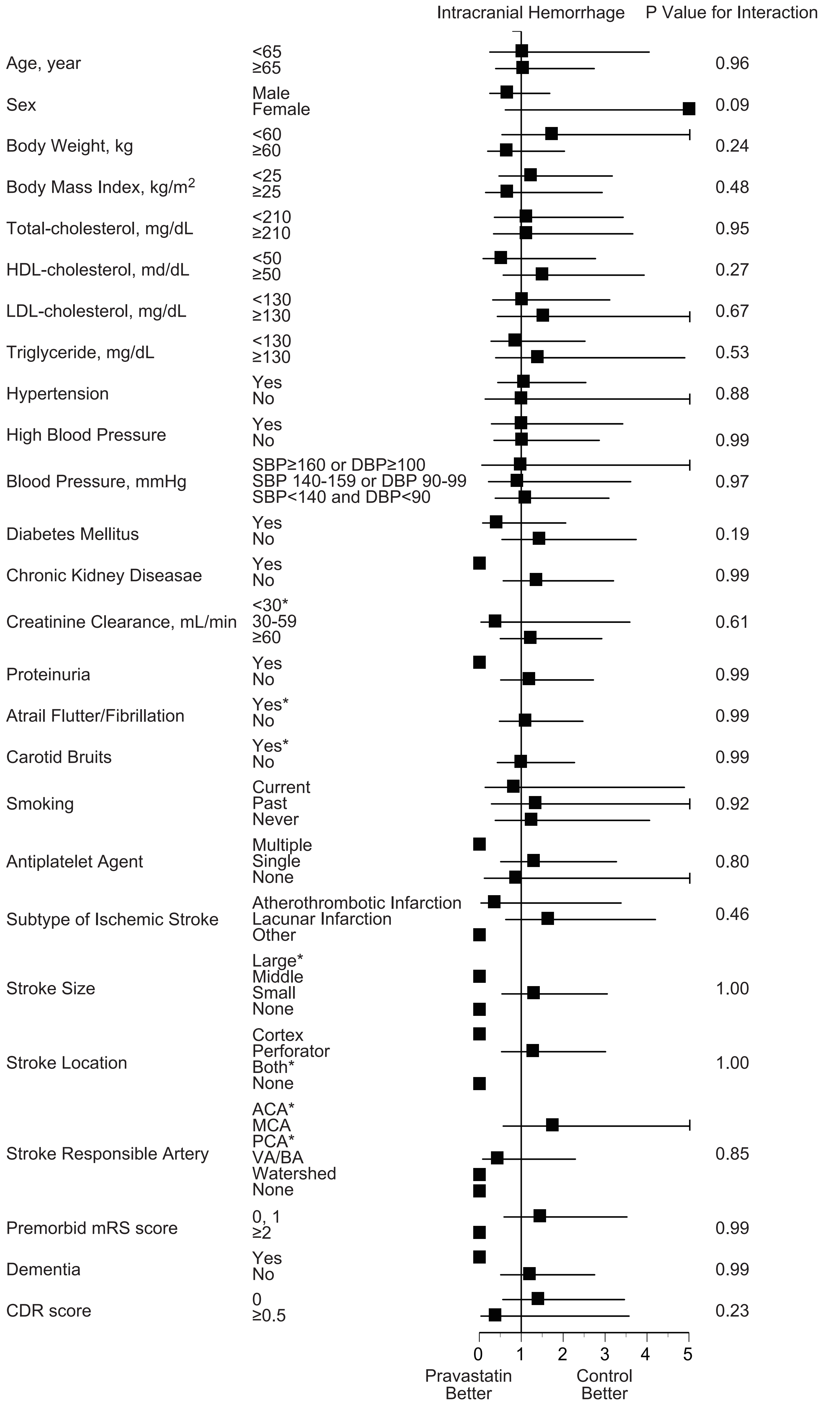

Supplementary Fig. S4. Cox proportional hazards for intracranial hemorrhage in pre-defined subgroups. Bars represent the relative risk with a 95%CI. P values for interaction test for heterogeneity of treatment across subgroups. *Hazard ratio was not obtained because of low number of subjects and events.

TIA, transient ischemic attack. HDL, high density lipoprotein. LDL, low density lipoprotein. SBP, systolic blood pressure. DBP, diastolic blood pressure. ACA, anterior cerebral artery. MCA, middle cerebral artery. PCA, posterior cerebral artery. VA, vertebral artery. BA, basilar artery. mRS, modified Rankin scale. CDR, clinical dementia rating.
