# Supplementary material for: The Japan Statin Treatment Against Recurrent Stroke (J-STARS): A Multicenter, Randomized, Open-label, Parallel-group Study
Source: eBioMedicine. 2015 Aug 6;2(9):1071–8. doi: 10.1016/j.ebiom.2015.08.006 (PMC4588424; doi:10.1016/j.ebiom.2015.08.006)
Supplement: Supplementary material. — Appendix. J-STARS Group: Organizational Structure and Participants. [file mmc2.pdf]

Appendix. J-STARS Group: Organizational Structure and Participants

Principal investigator: Masayasu Matsumoto MD, PhD

Professor & Chairman

Department of Clinical Neuroscience and Therapeutics, Hiroshima University Graduate School of Biomedical & Health Sciences

J-STARS central office: Tatsuo Kohriyama, Takemori Yamawaki, Toshiho Ohtsuki, Hiroshi Yamashita, Eiichi Nomura, Naohisa Hosomi, Kayoko Ishihara, Shiro Aoki, Tomohisa Nezu, Hisami Hashida, Yuki Kimura, Hitomi Takahashi, Hiromi Sumida, Chisato Masuda, Eri Kashima, Shiho Kamimoto, Kazuko Kojo.

Data Center: Masanori Fukushima, Yoji Nagai, Yoshihiro Matsubara, Tatsuo Kagimura, Satomi Sakabayashi, Takashi Kikuchi, Yoko Nakagawa, Kotone Matsuyama, Naoko Kashiwagi, Kyoko Murata, Shoko Oka, Shinya Iida, Hideki Kono.

Clinical Trial System Committee: Shotai Kobayashi.

Ethics Committee: Katsunori Kai.

Central Institutional Review Board: Kenji Kihira (Chair), Hiroaki Ikeda, Yoshiiku Kawakami, Katsushi Miyake.

Protocol Committee: Masanori Fukushima, Setsuro Ibayashi, Kazuo Minematsu, Yoji Nagai, Hideki Origasa, Shinichiro Uchiyama, Chiaki Yokota.

Preliminary Investigation Committee: Shotai Kobayashi (Chair), Setsuro Ibayashi, Katsumi Irie, Kazuo Minematsu, Eiichi Nomura, Norio Tanahashi.

Executive Committee: Setsuro Ibayashi, Toru Kita, Kazuo Kitagawa, Kazuo Minematsu, Makoto Takagi, Yasuo Terayama, Hideo Tohgi, Shinichiro Uchiyama.

Sub-executive Committee: Yutaka Furukawa, Jun Gotoh, Haruhiko Hoshino, Hidetaka Hougaku, Takanari Kitazono, Shu Konno, Tomomi Nakamura, Yukiko Tsutsumi, Haruko Yamamoto, Chiaki Yokota, Hisashi Yonezawa.

Independent Data Monitoring Committee: Takenori Yamaguchi (Chair), Yasuo Fukuuchi, Nobuo Hashimoto, Yuji Matsuzawa, Susumu Miyamoto (with the support by Yasushi Takagi), Hiroaki Naritomi, Eisei Oda, Yasushi Saito (with the support by Hideaki Bujo), Satoshi Teramukai.

Event Evaluation Committee: Shinichiro Uchiyama (Chair), Atsushi Hirayama, Izumi Nagata, Ryuji Nohara, Hiroshi Nonogi, Satoshi Okuda, Norio Tanahashi, Kazuo Yamada.

Analysis Committee: Hideki Origasa (Chair), Yoshihiro Matsubara, Tatsuo Kagimura.

Lipids Standardization Committee: Masakazu Nakamura.

Cognitive Function Standardization Committee: Etsuro Mori (Chair), Kenichi Meguro.

hsCRP Standardization Committee: Masakazu Nakamura (Chair), Kazuo Kitagawa.

Regional Promotion Committee: Yukito Shinohara (Chair); HOKKAIDO-TOHOKU DISTRICT- Kiyohiro Houkin, Ken Nagata, Jyoji Nakagawara, Akira Ogawa, Akifumi Suzuki, Hideo Tohgi; KANTO-KOSHINETSU DISTRICT- Yasuo Katayama, Yasuhisa Kitagawa, Norihiro Suzuki, Norio Tanahashi, Tamio Teramoto, Shinichiro Uchiyama; TOKAI-HOKURIKU DISTRICT- Shunro Endo, Masaru Kuriyama, Gen Sobue (with the support by Mizuki Ito), Kortaro Tanaka, Hidekazu Tomimoto, Kazuo Yamada, Hiroko Yamamoto; KINKI DISTRICT- Hidenao Fukuyama, Atsunori Kashiwagi, Kazuo Minematsu, Yasumasa Yamamoto, Toshiki Yoshimine; CHUGOKU-SHIKOKU DISTRICT- Genshi Egusa, Shotai Kobayashi, Shinji Nagahiro, Takeshi Shima, Michiyasu Suzuki, Shuhei Yamaguchi; KYUSHU DISTRICT- Yoichiro Hashimoto, Setsuro Ibayashi, Yasushi Okada, Jun Sasaki.

Investigators: HOKKAIDO-TOHOKU DISTRICT- Takenori Abe, Hitoshi Aizawa, Keiji Chida, Minoru Endo, Masashi Goto, Keiji Hara, Hiroko Ishikawa, Kenji Kamiyama, Taketo Kataoka, Takayuki Katayama, Takamasa Kayama, Yasuaki Kokubo, Shu Konno, Satoshi Koyama, Naohiko Kubo, Tetsuya Maeda, Yoshihiro Makita, Junichi Maruyama, Kazuo Mizoi, Ken Nagata, Jyoji

Nakagawara, Taizen Nakase, Hideki Ohyama, Toshiaki Osato, Tsukasa Saito, Kazuhiro Sasaki, Kenichi Sato, Shinya Sato, Tomohiko Sato, Jun Sawada, Yoshinobu Seo, Akifumi Suzuki, Hidekazu Takada, Yasuo Terayama, Hideo Tohgi, Toshiichi Watanabe, Toshiharu Yanagisawa, Hisashi Yonezawa; KANTO-KOSHINETSU DISTRICT- Kazunori Akaji, Hisanao Akiyama, Takekazu Akiyama, Takenori Akiyama, Shimon Amemiya, Nobuo Araki, Kazumasa Arii, Yasuhisa Daimon, Hiromi Eguma, Takaaki Fukuchi, Daisuke Furuya, Jun Gotoh, Makoto Hamamoto, Yasuhiro Hasegawa, Yukihiro Hasegawa, Akiko Hatano, Kenji Hiraga, Koichi Hirata, Tomo Horikoshi, Haruhiko Hoshino, Hisato Ikeda, Yukio Ikeda, Tatsuya Ishikawa, Toshihiko Iwamoto, Taro Kadowaki, Yutaka Kametsu, Nobuo Kamiya, Tadashige Kano, Takao Kanzawa, Yasuo Katayama, Toshiya Katsumata, Mitsuru Kawamura, Hiroaki Kimura, Kazuo Kitagawa, Yasuhisa Kitagawa, Akihiro Kiuchi, Yuichi Komaba, Shunichi Koyama, Akihiko Kuroiwa, Futaba Maki, Hiroshi Matsuda, Ban Mihara, Masahiro Mishina, Kazuko Mitani, Nobukazu Miyamoto, Kazuhide Miyazaki, Tohru Mizutani, Saori Moriya, Hidetomo Murakami, Kazuhiro Muramatsu, Takehiko Nagao, Takahide Nagashima, Tomomi Nakamura, Jiro Nakano, Kazunori Nanri, Minoru Nishiyama, Yasuhiro Nishiyama, Tomohide Ohnuki, Yuko Ohnuki, Sari Okamoto, Seiji Okubo,

Manabu Otomo, Satoru Otomo, Takashi Otomo, Makoto Sakurazawa, Naoshi Sasaki, Akira Sato, Tomokazu Shimazu, Takahiro Shimizu, Kikuko Shiobara, Makoto Shiraishi, Hiroshi Sugihara, Ryuji Suzuki, Kentaro Suzuki, Makoto Takagi, Youichi Takahashi, Masaki Takao, Masaru Takasaki, Hidetaka Takeda, Hidehiro Takekawa, Shunya Takizawa, Hideki Takubo, Norio Tanahashi, Ryota Tanaka, Yasutaka Tanaka, Yoshio Tanizaki, Sono Toi, Kentaro Tokuoka, Yutaka Tomita, Yukiko Tsutsumi, Shinichiro Uchiyama, Masayuki Ueda, Yuji Ueno, Takahiko Umahara, Takao Urabe, Hirofumi Watanabe, Koji Yamada, Hiroshi Yamaguchi, Kazuo Yamashiro, Kouhei Yamashita, Takashi Yasuda, Masayuki Yokochi, Yasuhiro Yoshii, Naoki Yuasa; TOKAI-HOKURIKU DISTRICT- Tetsuo Ando, Kenju Aoki, Shunro Endo, Akihiro Fujii, Youshi Fujita, Yuichiro Gondo, Hideo Hamada, Kensuke Hamada, Rina Hashimoto, Eri Hayakawa, Yumiko Hayashi, Mikio Hirayama, Genjirou Hirose, Kentaro Horibe, Masamichi Ikawa, Akira Inukai, Tomohiko Ishihara, Shinji Ito, Muichi Kaito, Takashi Kameyama, Kazuya Kawabata, Toshikazu Kawada, Keiko Kinomoto, Yasutaka Kobayashi, Ayuka Kubo, Masaru Kuriyama, Chiyuki Mabuchi, Tomoo Mano, Makoto Matsui, Masaru Matsui, Eiichirou Mukai, Kenya Murata, Shigemi Nagayama, Hiroto Nakagawa, Yoko Nakano, Tomoko Noda, Kazuya Nokura, Jun Ochiai, Hisashi Okada, Kenji Okita, Satoshi Okuda, Takafumi

Sagisaka, Toshimasa Sakakibara, Keishiro Sato, Mamiko Sato, Tatsuya Sato, Masaki Suenaga, Noriaki Suga, Shutaro Takashima, Shigeo Takeuchi, Takuya Tamura, Kortaro Tanaka, Shinichi Terao, Asako Ueno, Kimiko Umemura, Toshitaka Umemura, Hidetaka Watanabe, Kentaro Yamada, Hiroko Yamamoto, Osamu Yamamura, Keizo Yasui, Makoto Yoneda; KINKI DISTRICT- Hideo Aihara, Tsutomu Azuma, Hideki Etani, Jun Fujinami, Atsushi Fujita, Ryuzo Fukunaga, Hidenao Fukuyama, Naohiro Fumoto, Masahiro Funauchi, Shigetaka Furukado, Yutaka Furukawa, Masashi Hamanaka, Yoshiki Hase, Hiroshi Hasegawa, Kazuo Hashikawa, Masamichi Hayashi, Tachio Hikita, Shuichi Hirose, Hisanori Horiuchi, Taku Hoshi, Hidetaka Hougaku, Masafumi Ihara, Tadashi Ino, Haruhisa Inoue, Takashi Kasai, Tomonobu Kato, Hiromichi Kawai, Michi Kawamoto, Takeshi Kimura, Toru Kimura, Tomoharu Kinoshita, Toru Kita, Kazuo Kitagawa, Masataka Kitaguchi, Akihiro Kitamura, Junya Kobayashi, Mitsuo Kodama, Nobuo Kohara, Eiji Kohmura, Mika Komori, Kimito Kondo, Tomoyuki Kono, Eiji Kumura, Masato Kuroda, Kengo Maeda, Mari Matsumoto, Kazuo Minematsu, Toshiki Mizuno, Hidehiko Nabatame, Yoshinari Nagakane, Keiko Nagano, Kazuyuki Nagatsuka, Masanori Nakagawa, Shigenobu Nakamura, Yoshifumi Nakaya, Takehiro Nitta, Tomoyuki Ohara, Ryo Ohtani, Shinichiro Okamoto, Masaya Okamoto, Kenichi Oku, Emi

Omura-Matsuoka, Koh Ono, Kyoko Ozawa, Naoki Saji, Manabu Sakaguchi, Tsutomu Sasaki, Hirotaka Shimizu, Shun Shimohama, Toshiyuki Shiogai, Tetsuo Shioi, Rieko Suzuki, Masayasu Tabuchi, Masafumi Tagaya, Daisuke Takahashi, Makio Takahashi, Tsutomu Takahashi, Mikio Takaya, Tomohiro Tanaka, Haruhiko Terakawa, Hideo Terasawa, Kenichi Todo, Kazuki Tokumoto, Yasuhiro Tomii, Hidekazu Tomimoto, Kazunori Toyoda, Tetsuya Tsukahara, Hirokazu Ueda, Toshiyuki Uehara, Kiyohide Usami, Manabu Watanabe, Yoshiki Yagita, Hiroshi Yamagami, Tatsuhito Yamagami, Hodaka Yamakado, Shiro Yamamoto, Yasumasa Yamamoto, Hiroshi Yamauchi, Hitoshi Yasuda, Chiaki Yokota, Kenji Yoshikawa; CHUGOKU-SHIKOKU DISTRICT- Koji Abe, Yoshiaki Adachi, Tatsuo Akimura, Shiro Aoki, Atsuo Aoyama, Masamori Arai, Mutsuko Araki, Hirokazu Bokura, Kentaro Deguchi, Hiroki Fujii, Shunichiro Fujimoto, Toshihiro Fukusako, Akira Handa, Naoyuki Hara, Naoki Hayashi, Seiichiro Hibino, Jitsuo Higaki, Emi Hongo, Kie Honjo, Naohisa Hosomi, Shin-ichirou Ichihara, Keiji Igase, Yasuyuki Iguchi, Kenichi Iijima, Fusao Ikawa, Junko Ikeda, Naomi Ikuta, Eiji Imamura, Isao Inoue, Ken Inoue, Takeshi Inoue, Kayoko Ishihara, Kenichi Ishikawa, Hijiri Ito, Yuko Iwata, Yuishin Izumi, Ryuji Kaji, Tatsushi Kamiya, Yuhei Kanaya, Junichi Kanazawa, Takashi Kanda, Kenichi Kashihara, Satoshi Kataoka, Shinji Katayama, Shoichi

Kato, Motoharu Kawai, Kazumi Kimura, Juri Kitamura, Takeshi Kitamura, Katsuzo Kiya, Masuki Kobayashi, Shotai Kobayashi, Masakazu Kohno, Tatsuo Kohriyama, Hiroshi Kondo, Keita Kondo, Hisanori Kowa, Satoshi Kubo, Takashi Kurashige, Katsumi Kurokawa, Noboru Kusaka, Satomi Kushitani, Yasuhiro Manabe, Shinji Manabe, Hirofumi Maruyama, Ichiro Matsubara, Naoki Matsuoka, Hayato Matsushima, Nobuhisa Matsushita, Daisuke Matsuura, Chie Mihara, Yasuyo Mimori, Shingo Mitaki, Takafumi Miyachi, Michie Miyoshi, Ryo Mizuhara, Tomoko Muguruma, Tomoya Mukai, Yoshiki Murakami, Shinji Nagahiro, Masahiro Nagai, Yoshito Nagano, Hiroyuki Naito, Hiromitsu Naka, Minoru Nakagawa, Masahiro Nakamori, Takeshi Nakamura, Shunya Nakane, Kenji Nakashima, Hiroyuki Nakayasu, Takanori Nanba, Hisashi Narai, Takayuki Naya, Kiyoshi Negoro, Tomohisa Nezu, Kyoko Nishi, Shunjiro Nishide, Koichi Noda, Masahiro Nomoto, Eiichi Nomura, Kazuhide Ochi, Jun-ichi Ogasawara, Hiroaki Oguro, Takanori Ohnishi, Manabu Ohno, Tomohiko Ohshita, Toshiho Ohtsuki, Hideo Ohyama, Kazunori Okada, Kensho Okamoto, Bungo Okuda, Takafumi Okura, Nobuhiko Omori, Eiichi Onuki, Kunihiko Osaka, Naohiro Osaka, Sumio Ota, Yoko Ota, Hirokazu Sadahiro, Jun Saito, Hideki Sato, Kensaku Shibazaki, Takeshi Shima, Takeo Shishido, Yoshimasa Sueda, Tomohito Sugiura, Yoshihide Sunada, Norio Sunami, Yutaka

Suto, Michiyasu Suzuki, Yoshiteru Tada, Kazue Tajima, Kazuo Takahashi, Tetsuya Takahashi, Kazuhiro Takamatsu, Kensaku Takase, Ikuko Takeda, Nobuo Tamesa, Tetsuya Tamura, Hideo Terasawa, Yuka Terasawa, Hiromasa Toji, Hiroshi Tokinobu, Tsuyoshi Torii, Nobuyuki Umegae, Masaaki Uno, Hideaki Watanabe, Masao Watanabe, Atsuo Yamada, Shingo Yamagata, Shinya Yamaguchi, Shuhei Yamaguchi, Takuya Yamaguchi, Kanji Yamane, Hiroshi Yamashita, Takemori Yamawaki, Noboru Yokoyama, Takakazu Yokoyama, Hiroshi Yoneda, Ushio Yonezawa, Takeo Yoshimura, Kimihiro Yoshino, Junji Yoshioka; KYUSHU DISTRICT- Tetsuro Ago, Naoki Akamatsu, Shuji Arakawa, Masamichi Atsuchi, Masayuki Atsuchi, Kenichiro Fujii, Shigeru Fujimoto, Kenji Fukuda, Yoshihisa Fukushima, Haruyuki Goda, Makoto Goda, Toshiomi Goto, Yasuyuki Hara, Yoshiya Hashiguchi, Tomoyo Hashimoto, Yoichiro Hashimoto, Shigeaki Hayashi, Saho Higashi, Teruyuki Hirano, Setsuro Ibayashi, Tsuyoshi Imamura, Yoshitomo Ishii, Eiichi Ishikawa, Hiromi Ishikawa, Takao Ishitsuka, Yasuyuki Ito, Juro Jinnouchi, Masahiro Kamouchi, Yuka Kanazawa, Junji Kasuya, Kazuhiro Kishikawa, Jiro Kitayama, Takanari Kitazono, Masatoshi Koga, Yasuhiro Kumai, Masaya Kumamoto, Yoshikazu Maruyama, Ryu Matsuo, Hideki Matsuoka, Mayumi Mori, Kota Mori, Hiroyuki Murai, Makoto Nakajima, Hiroshi Nakane, Toshihisa Nakano, Toshiyasu Ogata,

Yasushi Okada, Tsuyoshi Omae, Hiroaki Ooboshi, Masato Osaki, Yoshisuke Saku, Tomohiko Sato, Junichi Takada, Kotaro Takamatsu, Kei-ichiro Takase, Nobuyoshi Takashima, Yuko Tanaka, Atsushi Tashima, Takahisa Tateishi, Tadashi Terasaki, Sadatoshi Tsuji, Akihiko Ueda, Takeshi Uwatoko, Kuniyasu Wada, Masayuki Wakita, Yoko Wakugawa, Yoshiyuki Wakugawa, Masaki Watanabe, Takeshi Yamada, Fumio Yamamoto, Masahiro Yasaka, Kiminobu Yonemura, Sohei Yoshimura.

Participating institutions (123): HOKKAIDO-TOHOKU DISTRICT (13); Nakamura Memorial Hospital, Asahikawa Medical University Hospital, Asahikawa Rehabilitation Hospital, Matsuzono Second Hospital, Morioka Municipal Hospital, Iwate Medical University, Japanese Red Cross Morioka Hospital, National Hospital Organization Iwate Hospital, Senseki-Hospital, Tohoku Pharmaceutical University Hospital, Research Institute for Brain and Blood Vessels-Akita, Akita University Hospital, Yamagata University Faculty of Medicine; KANTO-KOSHINETSU DISTRICT (23); Tokyo Saiseikai Central Hospital, Juntendo University Hospital, Nippon Medical School, Towa-Hospital, Showa University, Ebara Hospital, Tokyo Medical University, Tokyo Women's Medical University Hospital, Tama-Hokubu Medical Center, Tokai University

Hachioji Hospital, Tokyo Medical University Hachioji Medical Center, Nippon Koukan Hospital, Kawasaki Municipal Ida Hospital, St.Marianna University School of Medicine, Sagamihara Chuo Hospital, Tokai University Oiso Hospital, Tokai University School of Medicine, Saitama Medical University, Saitama International Medical Center, National Hospital Organization Saitama National Hospital, Nippon Medical School Chiba Hokusoh Hospital, Dokkyo Medical University, Institute of Brain and Blood Vessels Mihara Memorial Hospital; TOKAI-HOKURIKU DISTRICT (15); Toyama University Hospital, Toyama Prefectural Central Hospital, Kanazawa Medical University, Fukui General Clinic, University of Fukui Hospital, Aichi Saiseikai Rihabilitation Hospital, Nagoya Ekisaikai Hospital, Fujita Health University Banbuntane Hotokukai Hospital, Chubu Rosai Hospital, National Hospital Organization Nagoya Medical Center, National Hospital Organization Higashi Nagoya National Hospital, Japanese Red Cross, Nagoya Daini Hospital, Nagoya City University Hospital, Kasugai Municipal Hospital, Gifu Prefectural Tajimi Hospital; KINKI DISTRICT (22); Osaka Medical Center for Cancer and Cardiovascular Diseases, National Hospital Organization Osaka National Hospital, Japanese Red Cross Society, Osaka Red Cross Hospital, Japan Community Health care Organization Osaka Hospital, Hanwa Memorial Hospital, Osaka University Graduate School

of Medicine, National Cerebral and Cardiovascular Center, Japan Community Health care Organization Hoshigaoka Medical Center, National Hospital Organization Osaka Minami Medical Center, Pegasas Baba Memorial Hospital, Kobe University Graduate School of Medicine, Kobe City Medical Center General Hospital, Hyogo Brain and Heart Center at Himeji, Keishinkai Kyoto Takeda Hospital, Kyoto Second Red Cross Hospital, Kyoto Prefectural University of Medicine, Graduate School of Medicine Kyoto University, Rakuwakai Otowa Hospital, Kyoto Kizugawa Hospital, National Hospital Organization Kyoto Medical Center, Shiga University of Medical Science, Shiga Medical Center for Adults; CHUGOKU-SHIKOKU DISTRICT (33); Tottori Prefectural Central Hospital, Tottori University Hospital, Shimane University Hospital, Shimane Prefectural Central Hospital, Ohda Municipal Hospital, Okayama University Hospital, Kawasaki Medical School, Department of Stroke medicine, National Hospital Organization Okayama Medical Center, Okayama Rosai Hospital, Okayama Kyokuto Hospital, Brain Attack Center Ota Memorial Hospital, Mifukai Vi-hara Hananosato Hospital, Suiseikai Kajikawa Hospital, Hibino Hospital, Hiroshima Prefectural Hospital, Hiroshima University Hospital, National Hospital Organization Kure Medical Center, Yokoyama Hospital, Chugoku Rosai Hospital, Kure Kyosai Hospital, National

Hospital Organization Higashihiroshima Medical Center, Shakaihoken Shimonoseki Kosei Hospital, Ube Industries, LTD. Central Hospital, Yamaguchi University Graduate School of Medicine, Tokushima University, Tokushima Prefectural Central Hospital, Kagawa University School of Medicine, Osaka Neurosurgical Hospital, Kagawa Rosai Hospital, Ehime Prefectural Central Hospital, Sadamoto Hospital, Matsuyama Shimin Hospital, Ehime University Graduate School of Medicine; KYUSHU DISTRICT (17); Japan Labour Health Welfare Organization Kyushu Rosai Hospital, Steel Memorial Yawata Hospital, University of Occupational and Environmental Health, National Hospital Organization Kyushu Medical Center, National Hospital Organization Fukuoka Higashi Medical Center, Kyushu University, Japanese Red Cross Fukuoka Hospital, Iizuka Hospital, St.Mary's Hospital, Imari Arita Kyoritsu Hospital, Faculty of Life Sciences Kumamoto University, Japanese Red Cross Kumamoto Hospital, Kumamoto City Hospital, Almeida Memorial Hospital, Atsuchi Neurosurgical Hospital, National Hospital Organization Kagoshima Medical Center, Imakiire General Hospital.

Clinical Research Coordinators: HOKKAIDO-TOHOKU DISTRICT- Megumi Chiba, Ryo Fujii, Sadao Hatanaka, Masayuki Miura, Hiromi Sakashita, Kuniko

Watanabe; KANTO-KOSHINETSU DISTRICT- Yachiyo Abe, Masami Akuzawa, Atsushi Ibaraki, Chie Ishigaki, Hajime Nakamura, Michiyo Nakamura, Hiroyuki Sekiguchi, Tomoyo Sekiguchi, Atsuko Shiratori, Kaoru Wakabayashi, Tomoko Watanabe, Makoto Yamamoto; TOKAI-HOKURIKU DISTRICT- Junko Fujita, Sadanobu Ito, Keiko Kato, Junko Mochizuki, Kazuyo Motoyama, Kazuko Nonoyama, Kaoru Sawano, Kayoko Sumino, Kumiko Takahashi, Masao Yoshida, Yumi Yoshida; KINKI DISTRICT- Yoko Amino, Yuka Arima, Satoko Inoue, Shoko Kamiyoshi, Yoko Kimoto, Yuka Kitada, Hifumi Kitamura, Mari Koyama, Ryoko Maekawa, Reiko Manabe, Masami Matsumoto, Aki Morisaka, Emi Nagayama, Tomoko Nakagawa, Yukari Nakata, Kaori Niboshi, Mizuho Nishino, Midori Sakaguchi, Chiho Toda, Miyoko Yamamoto, Miduki Yokoyama; CHUGOKU-SHIKOKU DISTRICT- Tomoko Fukushima, Misato Hamada, Setsuko Hase, Hisami Hashida, Shiho Kamimoto, Tamiko Kanagawa, Eri Kashima, Sayuri Kimura, Yuka Kimura, Yuki Kimura, Shiho Kobayashi, Kazuko Kojo, Yuka Kondo, Shinji Kume, Chisato Masuda, Makiko Miyamoto, Takako Mizutani, Saori Mori, Yuko Moriyama, Kimiko Nanno, Mayumi Ohguro, Daiki Ousaka, Kanako Sogo, Syuzou Shinozuka, Namiko Taka, Akira Takagi, Hitomi Takahashi, Satoru Taniguchi, Jun Tsuchiya, Kumi Yonezawa, Satoe Yoshiura; KYUSHU DISTRICT- Masumi Inaba, Shiho

Sakai, Yoko Sonoda

; and the coordinators from EP-Mint co., Ltd., e-SMO.Inc, Sehma co., Ltd., Site Support Institute Co.,Ltd, SNBL Clinical Pharmacology Center, Ltd.
